# Group and individual stability of three parenting dimensions

**DOI:** 10.1186/1753-2000-5-19

**Published:** 2011-05-24

**Authors:** Tormod Rimehaug, Jan Wallander, Turid Suzanne Berg-Nielsen

**Affiliations:** 1 Regional Centre for Child and Adolescent Mental Health, Faculty of Medicine, Norwegian University of Science and Technology (NTNU), Norway; 2 Levanger Hospital, Nord-Trondelag Health Trust, Norway; 3 University of California, Merced, CA, USA

## Abstract

**Background:**

The Parental Bonding Instrument, present self-report version, (PBI-PCh) includes three scales, Warmth, Protectiveness and Authoritarianism, which describe three dimensions of current parenting. The purposes of this study were to (1) evaluate the true and observed stability of these parenting dimensions related to older children, (2) explore the distribution of individual-level change across nine months and (3) test potential parental predictors of parenting instability.

**Methods:**

Questionnaires were distributed to school-based samples of community parents of both genders (n = 150) twice, nine months apart. These questionnaires measured parenting, parental personality and emotional symptoms.

**Results:**

Based on 1) stability correlations, 2) true stability estimates from structural equation modeling (SEM) and 3) distribution of individual-level change, Warmth appeared rather stable, although not as stable as personality traits. Protectiveness was moderately stable, whereas Authoritarianism was the least stable parenting dimension among community parents. The differences in stability between the three dimensions were consistent in both estimated true stability and observed stability. Most of the instability in Warmth originated from a minority of parents with personality, childhood care characteristics and lower current parenting warmth. For the Protectiveness dimension, instability was associated with higher Protectiveness scores.

**Conclusions:**

True instability with all three self-reported parenting dimensions can occur across nine months in a community sample related to older children (7-15), but it may occur with varying degrees among dimensions and subpopulations. The highest stability was found for the Warmth parenting dimension, but a subgroup of "unstably cold" parents could be identified. Stability needs to be taken into account when interpreting longitudinal research on parenting and when planning and evaluating parenting interventions in research and clinical practice.

## Background

Parenting is a complex aggregation of everyday parental behaviors, cognitions, emotions, attitudes and values under multiple influences, influenced by transactions across time between parental, child and contextual factors [1-3]. This implies influence by both stable and variable sources, which is reflected in the conclusions of the only review or meta-analysis on parenting stability we have found, concluding that "... child rearing is simultaneously enduring and different..." [4]. This complicates the question of how stable parenting is over time. In our view, it implies that some specification relative to population, method, time frame and conceptual level is required when considering the stability of parenting. Furthermore, stability has numerous aspects. It can be addressed as maintained group level or distribution or the individual degree of stability. Whereas stability can also be addressed as the group mean-level developmental change across years, our focus here was restricted to stability and change across months, a time frame where significant group level changes in parenting dimensions are not likely.

Knowledge about the stability and change in parenting across months in the population is important general knowledge. Moreover, this information is imperative when examining change or differences in parenting related to selected *non-ordinary *conditions, such as life-stage changes, dramatic events, illness, treatment processes, and importantly, clinical trials. Changes in parenting observed under these types of conditions may in part result from the natural instability of parenting rather than the influence of those conditions.

The meta-analysis by Holden and Miller [4], excluded studies on non-ordinary conditions and found considerable differences in level and variation of stability across time depending on the study method, the parenting construct, the time frame and the subgroups examined. However, in the meta-analysis only six of the time stability studies (11%) involved children above eight years of age and half of these were based on observational methods rather than parent report. Only one of these studies examined time frames of one year or less, and the meta-analysis excluded the few studies involving fathers. None of the included studies investigated individual-level change. Thus, this study's combination of having a time frame of less than a year, assessing parenting of older children and including parent reports of both genders fills a gap in parenting stability research. The Holden review summarized a considerable number of studies on parenting stability, but the topic nearly faded away after 1999. In this introduction, we concentrate on studies after 2000.

### Conceptualization and Measurement of Parenting Dimensions

Conceptualizations of parenting may focus on specific daily parenting behaviors or parenting characteristics aggregated across time. *Parenting dimensions *are often used to characterize parenting behaviors by aggregated concepts that are relevant across ages and situations [5] and suitable for reports from parents and other family informants. Holden and Miller [4] found higher stability for more aggregated and parent-centered concepts than age-related and child-centered concepts. However, for older children the stability of parenting dimensions is still not well documented within moderate time frames.

Although there have been various specific conceptualizations of general parenting styles, a recent review [6] concluded that three main themes are present among styles: namely warmth, autonomy support and structure. Related to this general conclusion and based on factor analyses in multiple samples, Kendler [7] proposed three parenting dimensions represented by the scales Warmth, Protectiveness and Authoritarianism, when modifying the Parenting Bonding Instrument (PBI) from earlier work by Parker [8]. Whereas the PBI has been commonly used in parenting research (376 publications across 10 years, including 25 in 2009 according to the ISI - Web of Science), we have not located any reports of stability related to current parenting measured with the PBI. This leaves a gap regarding important characteristics of both this instrument and the concepts it measures.

The two traditional approaches to stability, general developmental stability (group mean-level change) and group differential continuity (stability correlations), are not sensitive to the degree and probability of individual-level stability. However, when change and stability are evaluated under uncommon conditions, for example, in clinical settings, individual change is highly relevant. However, individual-level change as an aspect of stability is largely unexplored in many areas of psychology [9]. We have found only one study on individual-level change in parenting, but this study included only toddlers [10]. Thus, data on individual-level change related to older children are lacking in parenting stability research.

According to Holden and Miller [4], parenting stability is largely the result of parental factors, including childhood care (parenting in the previous generation), adult personality, parenting experience and parent-child gender combinations. However, instability in parenting may instead reflect fluctuations in parental states, situational factors and child behaviors. According to Holden and Miller, long-term developmental change in parenting is largely the result of adaptations to child development [4]. A more recent study by Loeber et al. [11] documented developmental trajectories of parenting aspects as age-curves (6-18years). They also found small or no mean-level changes and stability correlations between .50 and .70 across one-year periods, depending on parenting concept and child age. In an older study by Krampen [12] (included in the review [4]), mothers reported 10-month stability correlations from .61 to .89. These two studies are the only ones we have found on parenting stability within a year related to older children. However, they focused on quite different behavioral categories (child-rearing practices and family interactions), and none of them examined individual-level change.

One Dutch and one American study showed similarity between mothers and fathers in parenting stability across nine years in 3-12 year olds [13] and across one year in toddlers [10], respectively. However, many parenting stability studies include only mothers [4]. Some studies have shown parent gender differences for some aspects of parenting that depend on culture and the organization of daily family life [14]. Thus, gender differentiation in research is needed and extrapolation between genders should not be trusted. Examination of parenting stability should include both parent and child genders.

Holden and Miller [4] emphasized that observational methods will tend to underestimate parenting stability. They also noted a general increase in parenting stability across child age. However, these conclusions were based on studies that confounded child age and method. Other researchers have found that parenting stability does not continue to increase with age among older children [11,15] which should motivate research specifically related to older children.

### The Phenomenon of Stability, Time-frames and Stability Indicators

Bugental, Johnston, New and Silvester [16] called for greater attention to the stability of psychological characteristics over and beyond the commonly evaluated test-retest reliability of instruments used to measure those characteristics. When stability is addressed, it is often confused, even equated, with reliability. The true stability of a phenomenon is often only implicitly assumed, and the observed stability characteristics of instruments are often ignored. Another problem is that stability studies are often based on non-representative samples (e.g., patients, people experiencing significant life events) that are not suitable as reference samples [4,16].

In this study, stability will be addressed primarily across moderate time frames of months or less than a year in which developmental mean-level change is expected to be minor, but true change at an individual level still may occur. Investigating stability will only be meaningful within time frames where true change is possible according to the theoretical assumptions of the characteristics in question. The time limits for true change are open to argument for each psychological phenomenon.

The time limits of true change in parenting are not clear, given the multitude of factors influencing parenting, ranging from fluctuating states and dynamic interaction processes to highly stable factors [1-3]. The change in some factors may occur quickly, even over a period of hours and days, but their influence on dimensional characteristics of a person's parenting may still lag and accumulate slowly. Related to younger children, true change in parenting is possible across weeks or months, even for dimensions of parenting [4]. We expect this to also be the case for older children, although the time frames of change and the degree of stability may differ. Challenges of parenting change with the age of the child [11] and previous research indicates that parenting stability also differs as the child ages [4]. However, only minor, mean-level changes have been reported over periods of less than a year for dimensional characteristics of parenting [11].

A time frame of months or less than a year is typical for naturalistic or experimental studies of change under non-ordinary conditions, whereas stability reference information is scarce related to these time-spans and the parenting of older children. Our study will attempt to fill some of this gap by addressing both group distribution stability and individual-level stability of parenting across nine months and focusing children at age 8 and above.

#### Observed group stability

Stability correlations are the usual method of evaluating group distribution stability or, more precisely, differential continuity. Mean-level change is not included in our study because it is assumed nonexistent in the moderate time frame of nine months used in this study. Stability indicators that describe observed stability are always attenuated by measurement error, but attempts have been made to estimate and evaluate true stability.

#### True stability

True stability is different from observed stability and instrument test-retest reliability. True stability focuses on real changes in the phenomenon, and is therefore more interesting from a theoretical viewpoint. The weakness of any observed stability indicator is that they will show a mixture of true change and the influence of retest unreliability (i.e., transient and random measurement errors) [17]. Therefore, statistical estimations of true stability require controlling for the influence of measurement error.

Group estimates of true stability were introduced by Spearman [18] in the form stability correlations *corrected for the attenuation from measurement error *(CAME). However, the vulnerability of this estimate to reliability overestimations and correlated errors has drawn criticism [19]. Measuring stability in structural equation modeling (*SEM*) estimating the regression between occasions while allowing for item auto-correlations represents an improvement related to this criticism [20].

#### Comparative framework

A less sophisticated but practically useful alternative to evaluate true stability, is the comparison of the observed stability of a given instrument to that of an instrument chosen as a benchmark [17]. A good candidate to use as a high stability benchmark would be personality traits, which based on theory and empirical data have relatively high stability among adults [21]. For further comparison, we also included the emotional symptoms of anxiety and depression as phenomena that presumably have moderate to low stability [22]. A comparative ranking of observed stability in a framework of several constructs may add further information about stability characteristics.

#### Individual-level stability

Stability correlations do not inform about the size or probability of individual change and do not reflect differences in individual-level change. The distribution of individual-level stability, also referred to as individual differences in stability, was calculated in our study as changes in standardi*z*ed scores (*z*-scores). Using standardized scores, several indicators can describe observed individual-level stability, and can be compared between scales using common criteria in a common metric. The distribution of absolute change in standardized scores reflects variation in individual instability, and its mean can be used as an indicator of central tendency stability. However, by introducing cut-points, probabilities for degrees of individual change regardless of change direction can be calculated (e.g. the probability for 'changed' or 'no change'). However, there are no established limits for such categorizations.

The only study known to us reporting individual-level change in parenting [10] calculated the Reliable Change index (RC) [23] from a change distribution and used RC as a cut-off limit for evaluating true individual-level change in the same distribution. However, using RC in this way overestimates normal stability, and is a circular approach that violates the assumption that the RC value should be calculated from a distribution of repeated measures representing random measurement error only [23]. Our alternative was to select limits defined by standardized scores as a metric (see later).

A benefit of examining the distribution of individual-level change is that it may reveal |subgroups indicated by unevenly distributed stability. A representative community sample must be expected to include a relatively low prevalence of individuals subjected to *non-ordinary *individual or family factors, events or adversities that could affect the stability of parenting. A low prevalence will not affect the main distribution of change considerably, but such variation will always create background "noise" in the analysis of systematic differences in clinical and research interventions. When such non-ordinary variation is more prevalent (as in at-risk- and disadvantaged populations), its extent and sources are more important to uncover.

Whereas predictors of stability or instability are not the primary aim of this study, their associations may also inform an evaluation of stability. If the observed instability of a phenomenon is related to a known factor, it is unlikely that the observed change is only the result of random or transient change. All factors that influence parenting may predict its stability [4], including personality traits, childhood care, adult parenting experience and emotional problems [24,25]. Therefore, in the present study, these influences are investigated together with age and gender as potential predictors of parenting stability.

### Aims

The primary aims of this study were (1) to evaluate the stability characteristics of the three parenting dimensions warmth, protectiveness and authoritarianism across nine months related to older children as expressed by (a) stability correlations, (b) true stability estimates and (c) the distribution of individual change, (2) to compare these stability characteristics to those of parental personality traits and emotional symptoms, (3) to examine associations between parenting instability and parents' gender, age, personality traits, previous generation parenting, parenting experience and emotional symptoms (anxiety and depression) to illuminate possible stability predictors and characteristics of stability subgroups.

## Methods

### Sample and Procedure

Parents were invited for Wave 1 from 20 randomly selected public schools in two counties. Of 558 eligible parents, 442 participated at the first time-point, T1. Half of them (*n *= 220) were randomly selected to participate again in Wave 2 nine months later for the purpose of this study, and 150 did so at the second time-point T2 (68% of those invited for Wave 2). No considerable differences were found between the Wave 2 participants, T2 dropouts or all those participating only in Wave 1. The nine-month time interval was chosen because it is suitable for investigating stability of parenting in a time frame without mean-level change and because it is comparable to the six to twelve months follow-up periods often chosen in clinic trials Questionnaires were distributed in closed envelopes to the children of participants who took them home from school, and they were returned by prepaid post. For the majority of children (68%), both a father and a mother completed the measures. The final sample at T2 included urban areas, small towns and rural districts, showing no significant differences in parenting scores. Parental age ranged from 26 to 58 years with a mean of 40.6 years (*SD *= 5.6), and 59% were mothers. Age of the children ranged from 8 to 15 years (*M *= 11.4, *SD *= 2.9), and their parents had 1 to 6 children, (*M *= 2.6, *SD *= 0.9).

The study was registered at the Norwegian Social Science Data Services and complied with the Helsinki Declaration. Approval was also obtained from the management of each of the schools for the study to be carried out in their respective schools, and written informed consent was secured from all parents by the school management.

### Instruments

Current *parenting *and *previous generation parenting *were measured in this study using Kendler's modification of the Parental Bonding Instrument (PBI) [7]. The modification reduced PBI to 16 items and constructed scales based on factor-analysis with varimax rotation. Factors with eigenvalues greater than unity were extracted into seven materials representing different informant positions. This construction procedure resulted in a strong three-factor solution independent of informant position, comprising the scales Warmth, Protectiveness and Authoritarianism [7]. These dimensions will be capitalized throughout this paper when referring to the PBI scales, but not when referring to them as concepts. The Warmth scale aggregates parenting characterized by positive emotions and empathic communication ("...talks with a warm and friendly voice..."), the Protectiveness scale comprises protection and infantilization ("...treat as younger..."), and the Authoritarianism scale covers parenting that restricts and directs the child ("...decide for him/her...") [7]. The self-report parent version asking about current parenting is referred to here as PBI-PCh. The offspring informant version asking adults about their retrospective childhood experiences of parenting is termed *previous generation parenting*, and describe separately the recalled maternal (PBI-M) and paternal (PBI-F) relationship (jointly referred to as PBI-M/F). Unless specified as *previous generation parenting*, the term 'parenting' throughout this paper refers to current parenting (PBI-PCh).

*Emotional symptoms *were measured with the Hospital Anxiety and Depression Scales (HADS), a self-report instrument of depressive and anxiety symptoms [26]. Separate scores are produced for Anxiety (A) and Depression (D) scales. With the exception of stability, the psychometric properties of these scales have been well documented [27]. Stability is only known in terms of movement in and out of "clinical caseness" (score ≥ 19) which showed considerable fluctuation across time for both anxiety and depression [22].

*Personality traits *were measured with a short-version of the NEO-PI [28], a measure of the "Big Five" personality traits (Neuroticism - N, Extraversion - E, Agreeableness - AE, Conscientiousness - C, Openness - O) with a highly replicable factor structure. The 100-item short-form of NEO-PI used here replicates the original factor structure and has corresponding high internal consistency for all five domains using 12 to 29 items for each domain [29]. The NEO-PI is used as a high stability benchmark. The literature is not consistent in identifying one NEO-PI dimension as having the highest stability, although Extraversion, Openness and Neuroticism are the primary candidates [21].

### Statistics

A comparison of the sampling groups in an unconditional random-effect regression effect model did not reveal significant sampling site contributions. Moreover, significant mother - father correlations within families were not found for any of the 16 instrument scales, confirming that a multilevel approach was not required.

The conversion of scales to standardi*z*ed *z*-scores was performed relative to gender and age distributions from the total T1 sample of this study (*N *= 442). Based on changes in *z*-scores, indicators of individual-level variation in stability were calculated. Lacking short-term test-retest values, cut-points were chosen based on Cohen's [30] recommendations for evaluating effect size, which propose *z *= .20, .50 and .80 as characteristic of small, moderate and large change in standardized group mean, respectively. Because our focus here is absolute individual change, which is more influenced by measurement error than group mean change, it was pertinent to set the lower limit for a considerably changed score at changes exceeding one standard deviation (i.e. absolute change Δz > 1.0) and calculating ***P***|Δ|>1*z *to represent its expectancy rate (denoted '*changed*' when referring to this definition). In a similar way one half of a standard deviation was chosen as an upper limit for negligible change, calculating the rate of T1-T2 differences smaller than 0.5 *z*-score as indicator (***P***|Δ|<0.5*z*, denoted '*no change*'). The rate of inter-mediate change ranging from 0.5 to 1.0 in absolute z-score change (***P***|Δ|0.5-1*z*) was included only for supplemental purposes (denoted '*uncertain change*').

The absolute change in z-scores (|Δ|*z*) was also used as a continuous variable in some analyses, and its mean (***M***|Δ|*z*) was calculated as a group stability indicator. The association between the categorization of absolute change ('no change' 'uncertain' and 'changed') and score level on both T1 and T2 was combined and tested as a between-subject effect in a T1-T2 repeated measures General Linear Model (GLM) in SPSS, with post-hoc Bonferroni contrasts between 'change' groups. To examine stability correlations between continuous variables, the Pearson product-moment correlation coefficient was used, denoted ***r ***for stability correlation and *r *for other correlations.

Using a comparative framework of other measures to evaluate observed stability requires that error-related psychometric properties of the included scales are acceptable and comparable. Especially important is scale unidimensionality in combination with scale internal consistency. These are estimated as the unidimensionality index *Comparative Fit Index *(*CFI*) and Cronbach's *alpha*. *CFI *was calculated in LISREL and considered acceptable if higher than .80, as recommended by Rogers et.al [31]. Because a low number of items reduces *alpha *significantly and the scales used here vary from four to 29 items, the average inter-item correlation (*r**^M^***) [32] has also been reported in Table 1. Unacceptable unidimensionality (*CFI*s < .80) in combination with reduced internal consistency and low inter-item correlations indicated scale construction problems for the Extraversion and Conscientiousness scales of this short version of the NEO-PI (see Table 1). Therefore, these two scales were excluded from further comparative analyses.

**Table 1:** True and observed stability indicators across 9 months (T1-T2) and internal consistency for current parenting, personality traits and emotional symptoms

	**T1 *alpha***	**T1 *r*^*M*^**	**T1 *CFI***	**T1-T2 *r^SEM ^*(*s.e*.)**	**T1-T2 *r***	**T1-T2 *M*|Δ|*z***	**T1-T2 *.P*|Δ|>1*z***	**T1-T2 *P*|Δ|<0.5*z***
	
*Current parenting (Parental Bonding Instrument -PBI-PCh)*								
Warmth	.77	.33	.98	.82 (.14)	.67	0.59	20%	62%
Protectiveness	.69	.31	.97	.69 (.14)	.58	0.69	24%	55%
Authoritarianism	.51	.21	.98	.62 (.14)	.49	0.77	29%	29%
*Personality traits (NEO-PI short version)*								
Neuroticism	.91	.27	.94	.87 (.12)	.86	0.39	6%	72%
Extraversion	.63	.11	.56	.85 (.18)	.69	0.63	20%	43%
Agreeableness	.86	.20	.82	.91 (.14)	.82	0.47	9%	64%
Conscientiousness	.72	.12	.61	.92 (.26)	.76	0.52	13%	57%
Openness	.82	.21	.84	.91 (.12)	.81	0.47	9%	60%
*Emotional symptoms (Hospital Anxiety and Depression Scales -ADS) *								
Anxiety	.80	.38	.97	.81 (.09)	.72	0.55	15%	53%
Depression	.72	.27	.97	.74 (.13)	.65	0.60	22%	62%

***r ***= stability correlations T1-T2, *alpha *= Cronbach's internal consistency alpha, *r**^M ^***= average inter-item correlation, *CFI *= Comparative Fit Index, ***r***^*SEM *^= true stability estimates in Structural Equation Modelling (*SEM*), *s.e*. = standard error of the ***r***^*SEM *^estimate, *M*|Δ|*z = *mean absolute change, ***P*|Δ**|>1*z *= rate of absolute change > 1.0z, ***P*|Δ**|<0.5*z *= rate of absolute change < 0.5z.

**Figure 1:**
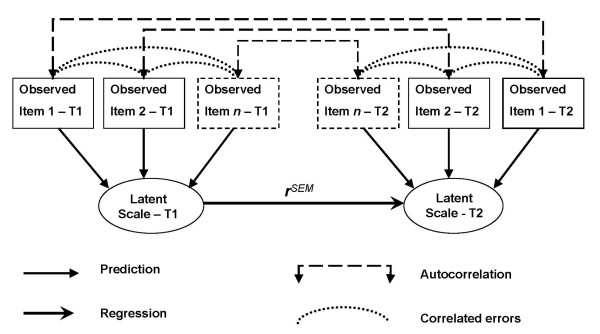
**Conceptual model for estimating true stability in structural equation modeling (*SEM*)**. The model estimates the regression term ***r***^*SEM *^between T1 latent scale and T2 latent scale based on the observed scores for scale items 1 to *n *at T1 and T2 respectively. Each of the eight scales Warmth, Protectiveness and Authoritarianism, Neuroticism, Agreeableness, Openness, Anxiety and Depression were estimated in separate models. The model allowed all item autocorrelations T1-T2, whereas allowing selected correlated item errors within T1 or T2 only when these increased model fit.

For true stability estimates, ***r^SEM ^***(*γ *regression term in LISREL output) were calculated in LISREL by regressing T2 on T1 latent scales in *SEM*, following procedures described by Jöreskog and Sörbom [20] and illustrated by the conceptual model in Figure 1. Calculations were performed separately for each of the eight subscales used in the comparative framework. The latent T1 and T2 scales were estimated from the respective T1 and T2 responses to items constituting the scale, allowing for T1-T2 item autocorrelations. In addition, selected error term correlations between items within T1 and T2 were allowed, only if these increased the model fit. This was the case for a smaller proportion of error term correlations (Warmth 4/43, Protectiveness 2/20, Authoritarianism 0/12, Neuroticism 20/812, Agreeableness 34/650, Openness 4/122, Anxiety 0/42, Depression 0/42). All eight estimation models produced fit indices *RMSEA *< .09, *RMR *< .09 and *CFI *>.93 (except the two previously excluded NEO-PI scales). The true stability (***r^SEM^***) estimation procedures resulted in confidence intervals ranging from .36 to .56 within the absolute range of 0 to 1.0. Because testing the statistical significance of differences in ***r^SEM ^***would have required a much larger sample, such tests were not performed here.

Difference in rates of 'changed' or 'no change' between scales were tested in one-sample binomial tests. Difference between stability correlations ***r ***were tested for statistical significance by converting each difference to a *z*-score relative to sample si*z*e (Fisher's transformation), and examining its probability as a *t*-test. This was calculated in Excel. When not otherwise specified, calculations and analyses were performed in SPSS 16.0.

Associations between potential predictors and individual-level *instability *in parenting dimensions, as expressed by the continuous variable of absolute change in z-score (|Δ|*z*) T1-T2, were examined with product-moment correlations between instability (|Δ|*z*) and predictors measured at both T1 and T2, but only those correlations replicated at both T1 and T2 were considered reliable and reported.

## Results

**Figure 2:**
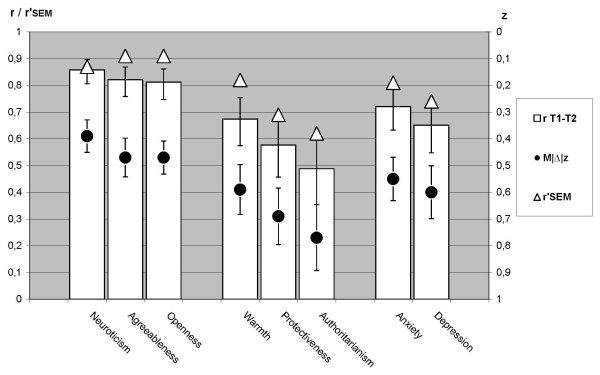
**Observed and true stability**. Observed stability correlations ***r ***(bars, with scale on the left) and mean absolute standardized change ***M***|Δ|*z *(black filled circles, with scale on the right) for each scale, with 95 percentile confidence intervals indicated for both. True stability estimates from *SEM *analyses are indicated with triangles. Both vertical scales are arranged with maximum stability at upper end.

**Figure 3:**
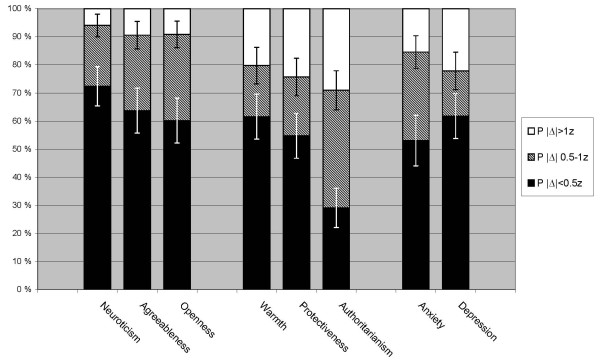
**Prevalence of 'changed' ***P***|Δ|>1*z *and prevalence of 'no change' ***P***|Δ|<0.5*z *with 95 percentile confidence intervals indicated for both, combined in cumulative bars with the intermediate 'uncertain change' ***P***|Δ|0.5-1*z *to illustrate distribution of individual stability**.

Observed stability correlations ***r ***and true stability estimates ***r^SEM ^***for all scales across nine months are reported in Table 1 together with the three *z*-based distributional characteristics of individual-level stability (***M***|Δ|*z*, ***P***|Δ|>1*z*, ***P***|Δ|<0.5*z*) and internal consistency *alpha*. The true stability estimates ***r^SEM^***, stability correlations ***r ***and the *z*-based indicators ***M***|Δ|*z *with confidence intervals are also illustrated in Figure 2. The prevalence of 'changed' scores (***P***|Δ|>1*z*) and 'no change' (***P***|Δ|<0.5*z*) are illustrated in Figure 3, which also includes confidence limits for these two rates and shows the intermediate 'uncertain change' proportion ***P***|Δ|0.5-1.0*z*. This intermediate proportion is informative primarily because a small proportion can indicate split distributions. Table 2 shows statistical tests comparing the stability of PBI parenting dimensions to the stability of personality traits and emotional symptoms.

**Table 2:** Differences in stability, compared pairwise between current parenting dimensions (columns) and personality traits or emotional symptoms (rows).

		Current self-reported parenting (Parental Bonding Instrument)		
	
		Warmth	Protectiveness	Authoritarianism
**Personality traits (NEO-PI)**				
			
**N**	Observed stability	**Δ*r *= -.19, *p *< .001**	**Δ*r *= -.28, *p *< .001**	**Δ*r *= -.37, *p *< .001**
	'Changed' ***P|Δ***|>1*z *	**+14%, *p *< .001**	**+18%, *p *< .001**	**+23%, *p *< .001**
	'No change'***P|Δ***|<.5*z*	**-10%, *p *< .010**	**-17%, *p *< .001**	**-43%, *p *< .001**
			
**A**	Observed stability	**Δ*r *= -.15, *p *< .01**	**Δ*r *= -.24, *p *< .001**	**Δ*r *= -.33, *p *< .001**
	'Changed' ***P|Δ***|>1*z*	**+11%, *p *< .010**	**+15%, *p *< .001**	**+20%, *p *< .001**
	'No change'***P|Δ***|<.5*z*	-2%, *ns*.	**-9%, *p *< .010**	**-35%, *p *< .001**
			
**O**	Observed stability	**Δ*r *= -.14, *p *< .01**	**Δ*r *= -.23, *p *< .001**	**Δ*r *= -.32, *p *< .001**
	'Changed' ***P|Δ***|>1*z*	**+11%, *p *< .010**	**+15%, *p *< .001**	**+20%, *p *< .001**
	'No change'***P|Δ***|<.5*z*	+2%, *ns*	-5%, *ns*.	**-31%, *p *< .001**

**Anxiety/Depression (HADS)**				
			
A	Observed stability	Δ***r ***= -.07, *ns*	**Δ*r *= -.14, *p *< .025**	**Δ*r *= -.21, *p *= .001**
	'Changed' ***P|Δ***|>1*z *	+5%, *ns*	+9%, *ns*	**+14%, *p *= .037**
	'No change'***P|Δ***|<.5*z*	+9%, *ns*.	.+2%, *ns*.	**-34%, *p *< .001**
			
D	Observed stability	Δ***r ***= +.02, *ns*	Δ***r ***= -.07, *ns*	**Δ*r *= -.16, *p *< .025**
	'Changed' ***P|Δ***|>1*z *	-2%, *ns*	+2.1%, *ns*	**+7%, *p *< .05**
	'No change'***P|Δ***|<.5*z*	-0.2%, *ns*.	-7.0%, *ns*.	**-32.6%, *p *< .001**

Observed stability correlations and the prevalence of individual-level change are compared separately.

NEO-PI = Big Five Personality Inventory (short version), HADS = Hospital Anxiety and Depression Scales, N = neuroticism, A = agreeableness, O = Openness, Δ***r ***= stability correlation difference, Δ**% **= rate difference - one-sample binomial test, *ns*. = non-significant, *p *= One-sided test of statistical significance, ***P|Δ***|>1*z *= rate of absolute change > 1.0z, ***P|Δ***|<0.5*z *= rate of absolute change < 0.5z

### PBI-PCh Stability Indicators

As shown in Table 1 and illustrated by Figure 2 and 3 the stability of the three parenting dimension scales was consistently ranked in the same order regardless of which indicators were used. Warmth showed the highest stability, Protectiveness intermediate stability and Authoritarianism the lowest stability among the three.

When testing for differences in stability between the parenting dimensions, only the contrast between Warmth and Authoritarianism reached statistical significance when evaluated by observed stability correlations ***r ***(Δ***r ***= .18, p < .01) and the probability for 'changed' scores (***P***|Δ|>1*z - *Δ***P ***= 9%, p < .01). The Protectiveness stability correlation was not significantly different from those of the other two dimensions. Additionally, for Authoritarianism, the 'no-change' probability (***P***|Δ|<0.5*z*), indicating very low stability, was significantly different from both Warmth and Protectiveness (Δ***P ***= 32% and 25%, p < .01), whose mutual difference was not significant.

### Comparative Framework

Personality traits had been chosen to represent high stability in the comparative framework. As shown in Table 2 all stability indicators used here showed higher stability for neuroticism than for any of the parenting dimension. For personality agreeableness and openness, only the 'no change' probability (***P***|Δ|>1*z*) deviated from this main pattern. Parenting warmth showed a split distribution of individual change in that 62% showed 'no change' and 20% showed 'changed' warmth (see Table 1). This split pattern was highly similar to the stability distribution characteristics of depression.

As shown in Table 2 the moderate stability revealed for Protectiveness, was clearly lower than that of personality traits. Protectiveness was only somewhat lower than depression or anxiety, only significantly different from the anxiety stability correlation, not for any aspect of individual-level change. In contrast, Authoritarianism was even less stable, indicated by the low 'no-change' rate ***P***|Δ|<0.5*z = *30% (lowest among all included scales) and the high rate of 'changed' scores ***P***|Δ|>1*z *= 29% (highest among all scales). The stability correlation of Authoritarianism, ***r ***= .49, was significantly lower than for all other scales, and the true stability estimate ***r*^*SEM *^**= .62 was lowest among all scales.

### Associations with Parenting Instability

Testing the association between individual-level 'change' categories and score level within dimensions showed that the most stable group for Warmth was characterized by significantly higher Warmth scores (*F*(2,140) = 5.97, *MSE *= 1.62, p = .003). The Bonferroni post hoc contrasts revealed significantly higher Warmth only in the contrast of the 'no-change' and 'changed' group (Δ*z *= +.65 cl95 ± .45, p = .003).

Instability in Warmth (|Δ|*z*) was negatively associated with NEO-PI Agreeableness (both *r*_1 _and *r_2 _*= -.25, p < .05), and NEO-PI Openness (both *r*_1 _and *r_2 _*= -.22, p < .05). Warmth instability (|Δ|*z*) was also negatively associated with previous generation maternal Warmth (*r*_1 _= -.17 and *r*_2 _= -.18, p < .05) but not to current parental emotional symptoms.

For Protectiveness, the most stable group was characterized by significantly lower Protectiveness scores (*F*(2,145) = 3.59, *MSE *= 1.60, p = .030). The Bonferroni post hoc contrasts revealed significantly lower Protectiveness only in the contrast between 'no-change' and the 'changed' group (Δ*z *= -.48 cl95 ± .43, p = .025). For Authoritarianism there were no reliable associations between stability categories and score levels.

The instability of Protectiveness and Authoritarianism was not associated with any of the potential parental predictors measured by PBI-M/F, NEO-PI(sv), or HADS. Child age or gender, parental age or gender, or parental experience (number of children) was not associated with the instability of any of the parenting dimensions.

### Supplementary analyses

Mothers reported significantly higher Warmth than fathers at both T1 and T2 by .06-.07 *SD *in a GLM analysis (F(1/146) = 19.85, *MSE *= 10.03, p < .001), but no difference for Protectiveness and Authoritarianism. Child gender was not significantly related to stability for any parenting dimension. All stability analyses were corrected for parent gender difference through conversions to gender-related *z*-scores.

The three parenting dimensions correlated only weakly (*r *= -.18 to +.32, *p *< .01). Moreover, their directional change T1-T2 and absolute change T1-T2 were not significantly correlated between dimensions. There was no mean-level change from T1 to T2 for any parenting dimension, and individual changes in either direction were equally frequent.

## Discussion

The three self-reported parenting dimensions exhibited different levels and patterns of stability over nine months in parents of older children (7 to 15 years). This general pattern of stability was consistent using all three statistical approaches to stability: estimated true stability, observed stability correlations and individual-level change, as illustrated in Figure 2 and 3.

Parenting warmth was rather stable; although not as stable as personality traits, it was similar to the stability of depressive symptoms. As with depressive symptoms, instability in warm parenting originated mainly from a subgroup consisting of 20% of the sample. Unstable warmth was associated with low personality trait scores on agreeableness and openness and with low childhood maternal warmth; however, it was not associated with current depressive symptoms.

Protectiveness was moderately stable, similar to stability in anxiety symptoms, whereas Authoritarianism showed lower stability than all of the other scales tested, although still in the lower moderate stability range.

Comparing our observed stability correlation for warmth (.67) to previous studies with older children, Krampen [12] found a higher stability correlation of .86 for emotional warmth, and Loeber found correlations around .69 for "bad relationship" [11]. The authoritarianism and protectiveness concepts from PBI are less easily compared to the concepts in these two other studies [11,12], but concepts associated with use of dominance and supervision tended to produce lower stability correlations than warmth in both studies.

These estimates and stability correlations for older children from our study and other studies [11,12] appear high compared to stability data reported in the meta-analysis by Holden and Miller [4]. However, in their meta-analysis, the dominance of observational studies that focus on more specific parenting behavior related to younger children can explain this difference.

Previous studies of parenting stability have varied considerably in levels of conceptualization, methods of investigation and child age [4,10,11,15]. However, differences in stability between parenting aspects were rarely addressed directly in discussions of stability, although such variation were often reported in the empirical results.

Converting our true stability estimates (***r*^*SEM*^**) into *R^2^*-values (as seen in Table 1) showed that true stability explains 67% of the variance in parental warmth, 48% for protectiveness and 38% for authoritarianism over nine months. We will argue that high stability requires at least 50% explained variance based on true stability estimates (correcting for measurement error) for trait-like parenting concepts. This leaves warmth as the most stable parenting dimension in our study relatively, whereas protectiveness and authoritarianism can best be characterized as high and low within the moderately stable range. This is consistent with our individual-level analyses, which showed that the observed stability correlations concealed considerable instability in protectiveness and especially in the authoritarianism dimension.

Considering the combined influence on parenting of parent, child and contextual factors with quite different stability, variation in stability between parenting dimensions may reflect different influences from stable and fluctuating factors [4]. Groups of parents with different contextual conditions, parent or child characteristics, may thus show corresponding differences in parenting stability. Community parents in Norway should be representative of parenting in a quite safe and advantageous context with relatively low prevalence of non-ordinary conditions.

### Dimension-specific patterns and associations with stability

The majority of parents (63%) showed highly stable scores on the warmth dimension, typically at a "warm" level. However, warmth tended toward a split stability distribution, as a subgroup of parents (20%) displayed instability and a "colder" mean score compared to stable parents. Instability in warmth was also associated to lower scores for agreeableness and openness as personality traits, and colder previous generation maternal relationship.

This split stability pattern between a majority and a dysfunctional minority is strikingly similar to that of depressive symptoms. Depressive symptoms are known for their fluctuations and recurrences in vulnerable subgroups in the population [33]. A less-clear split pattern of instability associated with high protectiveness scores was found, suggesting 'inconsistent overprotection'. No other parent or child variables were predictive of protectiveness instability.

Rather than being observed only in a sub-group, some instability in authoritarianism was widespread. Taken together, these results raise the question of whether child or contextual factors not evaluated here may identify subgroups of instability for protectiveness or authoritarianism.

Some of the observed stability of authoritarianism and, to some degree, protectiveness may be due to measurement errors indicated by reduced internal consistency. However, the stability is too low to be accounted for only by error. Furthermore, *alpha *for these two scales is deflated by a low number of items. Additionally, the scales of PBI and those three used from NEO-PI have similar average inter-item correlations and good unidimensionality (see Table 1), and the true stability estimates show the same pattern of stability between dimensions. Still, the conclusions must be treated with some caution due to the wide confidence intervals of the true stability estimates.

The few differences between fathers and mothers should probably be interpreted in relation to contemporary cultural trends in Norway that favor gender equality and fathers are highly involved in daily child care and -rearing [34]. The cultural values of gender equality may influence how parents report on their parenting. However, the relatively broad parenting dimensions may not capture more subtle gender differences in parenting.

The instability in authoritarianism may suggest influence from rather common but fluctuating factors, such as parental challenges arising from disputes over rules and privileges. This is consistent with the lack of associations between stability and fixed parental or child factors. An interpretation related to local cultural attitudes disfavoring authoritarianism in Norway [35] is also possible. These may leave authoritarian strategies as an underreported occasional practice rather than a stable parenting style among the majority of parents. Finally PBI Authoritarianism scale may be too sensitive to ordinary aspects of parenting authoritarianism, and less sensitive to more clinical important dysfunctional aspects.

Examining the distribution of individual-level stability added important nuances to the stability characteristics beyond the information provided by stability correlations. The combined picture produced by rates of 'changed', 'uncertain change' and 'no change' in individual-level stability could reveal whether instability is widespread or only present in a minority group. The distribution of individual change can also describe instability in terms that are more easily related to clinical practice and intervention research by directly stating, related to chosen criteria, how common changes might occur.

### Implications for clinical and research application

A cold relationship, especially in combination with restrictiveness or harshness, has long been considered a pathogenic parenting factor [36]. However, more recent research suggests that inconsistency in parenting, especially "love inconsistency" [37] is a more potent pathogenic factor than stable cold or authoritarian parenting [38,39]. Related to anti-social behavior in children, the importance of inconsistency was raised early [40] Our study shows an association between instability and cold parenting, and suggests that there is a danger of overlooking inconsistency of both parental warmth and protectiveness in assessing these dimensions unless they are evaluated across time. Furthermore, occurrences of authoritarian parenting on single occasions will be a weak clinical indicator because fluctuations are common in this dimension. Again, assessment over time will provide a better clinical picture.

Regarding parenting interventions targeting warmth and adequate use of authority, these stability results imply that long-term stabilization and consistency of improvement should be assured. Furthermore there is a need for differentiation between inconsistency and inadequate levels when addressing parenting factors as risks.

### Strengths and Limitations

The primary strength of this study was the comparison of results across different indicators of stability, which expands the traditional focus on group stability correlations with true stability estimates and individual-level stability characteristics. Another strength was that several dimensions of parenting were compared and evaluated in reference to other psychological characteristics. Finally, regarding instability predictors, only replications across T1 and T2 were considered reliable.

The primary weakness was that a larger sample would have allowed for more accurate estimates and reduced confidence intervals, especially for true stability estimates [17]. The age range of children in this study does not allow generalizations to be made about younger children or older adolescents. The use of self-reports on parenting could have resulted in some overestimation of stability. Thus, replication of the findings using other informants could prove interesting. However, Krampen [12] found higher stability in parenting with reports from teenage child informants than they did with in parent self-reports, showing that self-reports do not necessarily produce the highest stability indications.

Wide confidence intervals for the true stability estimates in *SEM *weaken the basis for strong conclusions, although these estimates lead to the same conclusions as those reached based on observed stability correlations and individual-level change.

Comparison of observed stability is complicated by the differences in internal consistency, suggesting a different influence from measurement error, especially for the authoritarianism scale. Some of these differences are related the low number of scale items, which tend to deflate *alpha *[32] although average item intercorrelations are rather similar for the PBI-PCh and the three included NEO-PI scales.

## Conclusions

The three parenting dimensions varied considerably in their stability across nine months among parents of older children. Although highly stable among the majority, change in warmth was observed in a subgroup of parents, resulting in lower stability than personality traits. In comparison, protectiveness was moderately stable, and authoritarianism appeared as the least stable dimension, although still in the lower moderate range. Thus, true fluctuations in self-reported parenting dimensions must be considered quite possible across months, even in ordinary samples, although the degree of change may depend on the parenting dimension and the selected population.

Even when using the PBI, which is based on parenting concepts approaching a trait level of aggregation, and assessing parenting over a relatively short time-span of nine months, none of the three parenting dimensions approached the stability level of personality traits. Rather, the parenting dimensions showed stability characteristics more similar to emotional symptoms like anxiety and depression, and even less stable.

Specifying influences of stability and change on each parenting aspect may be necessary to improve our understanding and ability to target parenting effectively in interventions. It is also important to bear in mind that although consistent warmth is optimal, protection and authority in parenting rather requires flexibility related to changes in child and contextual challenges. Adequate parenting related to these dimensions may require that parents pursue a dynamic rather than fixed balance between safety and expansion and between guidance and autonomy [4].

## Competing interests

The authors declare that they have no competing interests.

## Authors' contributions

TR conceived and planned the study, conducted literature review, data collection and data analysis, composed the initial draft of the manuscript and responded to reviewer revisions. JW contributed in writing the manuscript, especially the introduction and discussion. TSBN contributed to the selection of instruments, and to the interpretation and presentation of the study in writing the manuscript. All authors read and approved the final manuscript.
